# Pulmonary alveolar proteinosis and anemia may be associated with poor prognosis in patients with *IARS1* variants

**DOI:** 10.1186/s13023-025-03885-z

**Published:** 2025-07-09

**Authors:** Shu-Yuan Li, Yu-Ting Wang, Teng Liu

**Affiliations:** 1 The Department of Gastroenterology, National Clinical Research Center for Child Health and Disorders, Ministry of Education Key Laboratory of Child Development and Disorders, Chongqing Key Laboratory of Child Neurodevelopment and Cognitive Disorders, Children’s Hospital of Chongqing Medical University, 136 Zhongshan Second Road, Chongqing, 400015 China; 2 The Center for Pediatric Liver Diseases, Children’s Hospital of Fudan University, 399 Wanyuan Road, Shanghai, 201102 China

**Keywords:** Anemia, Hepatopathy, Isoleucyl-tRNA synthase 1, Prognosis, Pulmonary alveolar proteinosis

## Abstract

**Background:**

Growth retardation, impaired intellectual development, hypotonia, and hepatopathy (GRIDHH) is a rare disease caused by compound heterozygous variations in the isoleucyl-tRNA synthetase 1 (*IARS1*) gene. To date, only a few cases have been reported and there has been no comprehensive analysis of its clinical, pathological, molecular genetic features, or factors associated with a poor prognosis.

**Methods:**

Three new cases of IARS1 deficiency have been documented. A review and summary of the clinical, pathological, and molecular genetic features of previously reported cases was conducted. The prognostic significance of identified risk factors was evaluated using Kaplan-Meier plotter analysis.

**Results:**

The 3 new cases harbored 6 novel variants in IARS1. The principal clinical manifestations of IARS1 deficiency were intrauterine growth retardation (13/13), failure to thrive (13/14), feeding difficulties (10/14), elevated aminotransferases (11/14), cholestasis (8/14), acute liver failure (7/14), hepatomegaly (7/14), hypoalbuminemia (10/14), coagulation abnormalities (8/14), microcephaly (11/14), neurodevelopmental delay (10/14), hypotonia (9/14), impaired intellectual development (6/7), recurrent infections (9/14), special facial appearance (8/14), zinc deficiency (4/7), and pulmonary alveolar proteinosis (3/14). The principal pathological features of the liver were fibrosis (6/8), hepatocellular steatosis (5/8), and cholestasis (5/8). A total of 24 variants were identified in 14 cases, comprising a frameshift variant (*n* = 3), nonsense variant (*n* = 3), splice variant (*n* = 2), and missense variant (*n* = 16). Of the 14 cases, five resulted in death. Kaplan-Meier analysis indicated that the occurrence of pulmonary alveolar proteinosis (HR = 10.837, 95% CI = 1.246–94.257, *P* = 0.031) and anemia (HR = 15.411, 95%CI = 2.101-113.057, *P* = 0.007) were associated with a poor prognosis.

**Conclusions:**

In this report, we present three new cases of IARS1 deficiency and provide a comprehensive summary of the clinical, pathological, and molecular genetic characteristics observed in all previously reported cases. Furthermore, our findings suggest that the presence of pulmonary alveolar proteinosis and anemia may be associated with a poor prognosis.

**Supplementary Information:**

The online version contains supplementary material available at 10.1186/s13023-025-03885-z.

## Background

Aminoacyl-tRNA synthetases (ARSs) constitute a family of enzymes that exhibit a high degree of conservation and are widely expressed in multiple organs. They are responsible for the specific attachment of amino acids to their corresponding tRNA molecules in the cytoplasm and mitochondria [1, 2]. Isoleucyl-tRNA Synthetase 1 (IARS1), a member of the ARS family, has been demonstrated to catalyze the attachment of isoleucine to its cognate tRNA. The gene encoding *IARS1* (OMIM *600709) is located on chromosome 9q22.31 and is comprised of 34 exons. Variants in the *IARS1* gene have been linked to a spectrum of clinical manifestations, including growth retardation, impaired intellectual development, hypotonia, and hepatopathy (GRIDHH; OMIM #617093). This was initially documented by Kopajtich et al., [3] with subsequent reports emerging in subsequent years [4–10]. 

This report presents three new cases of IARS1 deficiency and provides a comprehensive summary of the clinical, pathological, and molecular genetic characteristics observed in all previously reported cases. Furthermore, factors that may be associated with patient prognosis were evaluated.

## Methods

The inclusion criteria were defined as rare biallelic variants in *IARS1*, classified as pathogenic or likely pathogenic in accordance with the American College of Medical Genetics and Genomics (ACMG) guidelines for the interpretation of sequence variants [11]. A whole exome sequence was conducted to exclude other potential etiologies of the condition.

A search of the published literature was conducted using the databases PubMed and Wanfang Data Knowledge Services Platform. The search terms employed were “isoleucyl-tRNA synthetase”, “isoleucyl-tRNA synthetase 1”, “IARS”, “IARS1” and “GRIDHH”. Trials lacking patient information were excluded from further analysis.

### Liver histopathology

Liver biopsy specimens were fixed in formalin, routinely embedded in paraffin, and sectioned at 4 μm. Tissue sections picked up on glass slides were stained, using standard procedures, with hematoxylin and eosin, with the periodic acid-Schiff technique, with the Masson trichrome technique, for iron, and for reticulin fibers. Light microscopy was undertaken.

### Pathogenicity of newly discovered *ARS1* variants

Predicted pathogenicity of novel variants was assessed by the in silico tools Mutation Taster (http://www.mutationtaster.org/), Sorting Intolerant From Tolerant (SIFT, http://sift.jcvi.org), Protein Variation Effect Analyzer (PROVEAN, http://provean.jcvi.org/index.php), Mendelian Clinically Applicable Pathogenicity (http://bejerano.stanford.edu/MCAP/), Functional Analysis Through Hidden Markov Models (http://fathmm.biocompute.org.uk/), and Rare Exome Variant Ensemble Learner (https://sites.google.com/site/revelgenomics/). All were used with default settings.

### Statistical analysis

The statistical analysis was conducted using MedCalc medical statistics software (version 22.001). Student’s t-test was performed when data showed a normal distribution. Nonparametric Mann-Whitney U testing was performed when data did not show a normal distribution. Data are expressed as mean ± standard deviation or median (interquartile range, IQR). Fisher’s exact test is used in the “R×C table”. Kaplan-Meier plots were employed to evaluate potential prognostic factors, and discrepancies in survival were assessed using the log-rank test. A *P*-value of less than 0.05 was deemed to be statistically significant.

## Results

### Case description

Three children were admitted for further investigation, due to a suspicion of cholestasis and/or cough caused by IARS1 deficiency. The three children were enrolled in the study with the consent of their parents, in accordance with a protocol approved by the Children’s Hospital of Chongqing Medical University and the Children’s Hospital of Fudan University. The study was conducted in accordance with the ethical guidelines set forth in the Declaration of Helsinki (1975).

#### Case 1

Case 1 is a 2.5-month-old male infant who was admitted to the hospital with a cough and jaundice of the skin, which persisted for a period of 1.5 months. The infant was delivered via caesarean section at 37 + 3 weeks gestation with a birth weight of 1.70 kg, which is below the third percentile for gestational age. Furthermore, the infant displayed cyanotic asphyxia at the time of birth. From the age of one month, the patient was admitted to the hospital on multiple occasions due to edema and coughing. Upon admission, the patient was diagnosed with cholestasis, growth retardation, anemia and refractory hypoalbuminemia. Despite the administration of antibiotics and ursodeoxycholic acid, the patient’s condition remained unimproved. The child was the second-born to non-consanguineous parents of Han Chinese descent. The first child, a female, was eight years of age and in good health. The parents denied a family history of liver disease and inherited metabolic disorders. A physical examination was conducted. The infant’s weight<3rd percentile (2.7 kg), height<3rd percentile (52 cm), head circumference<3rd percentile (33 cm). His respiration rate was 50 times per minute, and he exhibited no inspiratory triple concave sign. The subject displayed a listless demeanor, was malnourished, and exhibited a chubby face. Moderate jaundice was observed in the skin, and the presence of sputum sounds was noted in both lungs. The abdomen was observed to be soft, with the liver located at a depth of approximately 3.0 cm below the ribs and the spleen situated at a depth of approximately 1.0 cm below the ribs. The neurological examination yielded no obvious abnormal results.

The laboratory examination (Supplement Table 1) revealed anemia (hemoglobin: 72.0 g/L (normal range (NR), 99–196 g/L)) and a decreased serum-bound bead protein concentration of less than 0.058 g/L (NR, 0.3–2.1 g/L). The biomarker values for the hepatobiliary system exhibited elevated levels of total bilirubin (TB, 116.0umol/L (NR, 3.4-11.1umol/L)), direct bilirubin (DB, 103.1umol/L (NR, 0-6umol/L)), alanine aminotransferase (ALT, 66U/L (NR, 8-71U/L)), aspartate aminotransferase (AST, 109U/L (NR, 21-80U/L)), and total bile acids (TBA, 380.4umol/L (NR, 0-10umol/L)). Furthermore, there was a notable decline in gamma-glutamyl transpeptidase (GGT, 138.0U/L (NR, 9-150U/L)) levels in conjunction with hypoalbuminemia (25.4 g/L (NR, 35–50 g/L)), hypofibrinogenemia (0.5 g/L (NR, 2–4 g/L)), abnormalities of coagulation (international normalized ratio (INR): 2.4 (NR, 0.8–1.2), subsequent to vitamin K1 injection), hypoglycemia(1.6mmol/L (NR, 3.9-6.1mmol/L)), and normal blood ammonia. Additionally, the patient’s serum samples tested negative for IgM/IgG antibodies to Toxoplasma gondii, herpes simplex virus, rubella virus. The serum samples tested negative for HBsAg, anti-HBs, HBeAg, anti-HBe, anti-HBc, anti-HCV, antibodies to syphilis and human immunodeficiency virus. The urine samples revealed no evidence of bacterial and fungal contamination. Moreover, the results of the respiratory virology tests, glucose-6-phosphate dehydrogenase enzyme activity, Coombs tests, and blood tandem mass spectrometry were all within the normal range. The chest X-ray (Supplement Figure S1.A) revealed evidence of pneumonia, characterized by observable interstitial alterations. The abdominal ultrasound demonstrated an enlarged liver, extending 3.2 cm below the ribs, with increased parenchymal echogenicity, and a slightly enlarged spleen, extending 1.4 cm below the ribs. An ultrasound examination of the heart revealed the presence of a type II atrial septal defect with a diameter of 7 mm. The results of the brainstem auditory evoked potentials indicated the presence of abnormalities in the distal auditory nerves or cochlea bilaterally, with elevated bilateral hearing thresholds. Subsequently, whole exome sequencing (WES) was conducted on the proband’s DNA sample at Beijing MyGenostics Inc. (Beijing, China) using the GenCap Exome Capture Kit (MyGenostics Inc.). Exome sequencing analysis identified biallelic variants in the *IARS1* gene, specifically [c.2017–2 A > G (splicing)] was paternally inherited, and [c.241 A > G (p. Arg81Gly)] was maternally inherited. The results were validated by Sanger sequencing.

**Table 1:** Demographics and clinical manifestations of IARS1 deficiency in 14 patients

ID	G	Descent	Allele 1	Allele 2	Gestational age (weeks)	Growth	Nervous system	liver	Blood system	Musculature	respiratory system	endocrine system	Others	Status at last follow-up	Ref
1	M	German	c.[1252 C > T]; p.[Arg418*]	c.[3521T > A]; p.[Ile1174Asn]	38	IUGR, FTT	Neurodevelopmental delay, Intellectual disability, Delayed speech and language development, Microcephaly, bilateral spasticity	-	-	Muscular hypotonia	-	low levels of growth hormone-dependent factors IGF1 and IGFBP3	Feeding difficulties, Hypoalbuminemia, Recurrent infections, Decreased serum zinc, Strabismus, Esophagitis	Alive at 18.7y	[3]
2	F	Japanese	c.[760 C > T]; p.[Arg254*]	c.[1310 C > T]; p[Pro437Leu]	38	IUGR, FTT	Neurodevelopmental delay, Intellectual disability, Syncope, Hearing abnormality, Seizure	Elevated hepatic transaminase, Liver biopsy(2y): steatosis, portal-tract fibrosis	-	-	-	growth hormone deficiency, Insulin-dependent diabetes	Decreased serum zinc	Alive at 21y	[3]
3	M	Austrian	c.[1109T > G]; p.[Val370Gly]	c.[2974 A > G]; p.[Asn992Asp]	38^+ 4^	IUGR, FTT	Neurodevelopmental delay, Microcephaly	ALF, Cholestasis, Elevated hepatic transaminase, Abnormality of coagulation. Liver biopsy(14 m): steatosis, cholestasis, portal-tract fibrosis	-	Muscular hypotonia	-	-	Feeding difficulties, Hypoalbuminemia, Recurrent infections, Decreased serum zinc, Specific facial appearance	Alive at 3y	[3]
4	M	Israeli Arab	c.[2215 C > T]; p.[Arg739Cys]	c.[1667T > C]; p.[Phe556Ser]	37	IUGR, FTT	Neurodevelopmental delay, Microcephaly, Joint hyperlaxity	ALF, Cholestasis, Elevated hepatic transaminase, Abnormality of coagulation, Hyperammonemia Liver biopsy(1 m): cholestasis, Fibrosis with new vessel formation, bile ductular proliferation	-	-	-	abnormal IGF1 generation test	Feeding difficulties, Hypoalbuminemia, Specific facial appearance increased levels of 25- hydroxy vitamin D, Hydronephrosis	Alive at 4y	[4]
5	M	Polish	c.[2011del]; p.[Gln671fs]	c.[206 C > T]; p.[Thr69Ile]	39	IUGR, FTT	Neurodevelopmental delay, Intellectual disability, Delayed speech and language development	Elevated hepatic transaminase, Hepatosplenomegaly Liver biopsy(18 m): steatosis	-	Muscular hypotonia	-	-	Feeding difficulties, Recurrent infections, Decreased serum zinc, Specific facial appearance Autism	Alive at 7y	[5]
6	F	the Netherlands	c.[1305G > C]; p.[Trp435Cys]	c.[3377dup]; p.[Asn1126fs]	Full term	IUGR, FTT	Microcephaly	ALF, Cholestasis, Elevated hepatic transaminase, Abnormality of coagulation, hepatomegaly Liver biopsy(4 m): steatosis, cholestasis, extensive fibrosis	Anemia, Thrombocytosis Leukocytosis	Muscular hypotonia	pulmonary alveolar proteinosis	-	Feeding difficulties, Hypoalbuminemia, Recurrent infections Decreased serum zinc (/)	Dead at 0.33y	[6]
7	M	the Netherlands	c.[1305G > C]; p.[Trp435Cys]	c.[3377dup]; p.[Asn1126fs]	Full term	IUGR, FTT	Neurodevelopmental delay, Intellectual disability, Microcephaly	Elevated hepatic transaminase, Hepatomegaly, Abnormal liver sonography	Anemia, Thrombocytosis Leukocytosis	Muscular hypotonia	pulmonary alveolar proteinosis	-	Feeding difficulties, Hypoalbuminemia, Recurrent infections, Autism Decreased serum zinc (/)	Dead at 6y	[6]
8	M	Bangladeshi	c.[290 A > G]; p.[Asp97Gly]	c.[290 A > G]; p.[Asp97Gly]	Full term	IUGR(/)	Microcephaly	ALF, Cholestasis, Elevated hepatic transaminase, Abnormality of coagulation, hepatomegaly, Abnormal liver sonography, Liver biopsy(9y): hydropic degeneration of hepatocytes	/	-	-	-	inflammatory bowel disease	Alive at 9y	[7]
9	F	Chinese	c.[1604 A > G]; p.[Tyr535Cys]	c.[1604 A > G]; p.[Tyr535Cys]	Full term	IUGR, FTT	Neurodevelopmental delay, Intellectual disability, Delayed speech and language development, Microcephaly	Abnormal liver sonography	-	Muscular hypotonia	-	Abnormal thyroid hormone	Recurrent infections, Specific facial appearance, Cryptorchidism, Pica Decreased serum zinc (/)	Alive at 6y	[8]
10	F	Chinese	c.[701T > C]; p.[Leu234Pro]	c.[1555 C > T]; p.[Arg519Cys]	37^+ 4^	IUGR, FTT	Neurodevelopmental delay, Delayed speech and language development, Microcephaly, Joint hyperlaxity	ALF, Cholestasis, Elevated hepatic transaminase, Abnormality of coagulation, Hyperammonemia, Hepatosplenomegaly, Abnormal liver sonography	/	Muscular hypotonia	-	-	Feeding difficulties, Hypoalbuminemia, Specific facial appearance, Diarrhea	Dead at 1.6y	[9]
11	F	Chinese	c.120-1G > A; [splicing]	c.2164 C > A;p.Arg722Ser	Full term	IUGR, FTT	Neurodevelopmental delay, Microcephaly, Seizures, Developmental regression	-	-	Muscular hypotonia	-	-	Hypoalbuminemia, Specific facial appearance Recurrent infections Decreased serum zinc(/)	Alive at 1.4y	[10]
12	M	Chinese	c.[2017–2 A > G]; [splicing]	c.[241 A > G]; p.[Arg81Gly]	37^+ 3^	IUGR, FTT	Microcephaly, Hearing abnormality	ALF, Cholestasis, Elevated hepatic transaminase, Abnormality of coagulation, Hepatosplenomegaly, Abnormal liver sonography	Anemia	-	-	-	Feeding difficulties, Recurrent infections, Hypoalbuminemia, Specific facial appearance Decreased serum zinc(/)	Dead at 0.25y	Our study
13	M	Chinese	c.[2354del]; p.[K785Rfs*3]	c.[1556G > A]; p.[Arg519His]	38	IUGR, FTT	Microcephaly, Delayed speech and language development	Cholestasis, Elevated hepatic transaminase, Abnormality of coagulation, hepatomegaly, Hypoglycemia Hyperammonemia, Liver biopsy(2 m): cholestasis, portal-tract fibrosis, bile ductular proliferation, multinucleated giant liver cells	Anemia	-	-	-	Hypoalbuminemia, Specific facial appearance Decreased serum zinc(/)	Alive at 2.6y	Our study
14	M	Chinese	c.[1330 C>T]; p.[Arg444X]	c.[443 A > G]; p.[Tyr148Cys]	35^+ 4^	IUGR, FTT	Neurodevelopmental delay, Intellectual disability, Delayed speech and language development, Microcephaly	ALF, Cholestasis, Elevated hepatic transaminase, Abnormality of coagulation, hepatomegaly, Hypoglycemia Hyperammonemia	Anemia, Thrombocytosis Leukocytosis	Muscular hypotonia	pulmonary alveolar proteinosis	-	Feeding difficulties, Hypoalbuminemia, Recurrent infections, Decreased serum zinc(/) gastric volvulus	Dead at 3.1y	Our study

Abbreviations: G, gender; M, male; m, month; F, female; y, year; Ref, referenced publication; IUGR, intrauterine growth restriction; FTT, failure to thrive; /, data not available or data not reported; -, absent; ALF, acute liver failure; IGF1, insulin-like growth factor-1; IGFBP3, Insulin-like growth factor binding protein-3

The infant was administered a lactose-free, high medium-chain triglyceride (MCT) formula via nasogastric feeding tube, ceftazidime, and ursodeoxycholic acid, one red blood cell suspension transfusion, and multiple albumin supplements. However, his condition continued to deteriorate. His mental status declined, and he displayed poor responsiveness, malnutrition, feeding difficulties, an evident cough, and respiratory distress. Moreover, the patient’s jaundice worsened, and he also exhibited refractory anemia, hypoalbuminemia, hypoglycemia and coagulation dysfunction. In view of these, critical, conditions, the child’s parents made the difficult decision to cease further medical treatment. Following a 14-day period of hospitalization, the child was discharged without further intervention. Twenty days later, at the age of three months, the child died at home.

#### Case 2

Case 2 is a two-month-old male infant who was admitted to the hospital with a history of jaundice of the skin for a period of approximately six weeks. The jaundice of the skin commenced without any apparent cause and subsequently worsened, accompanied by the presence of light-colored stools. No additional symptoms were observed, such as fever, cough, or convulsions. The infant had been delivered after 38 weeks of gestation via caesarean section due to an abnormal fetal heart rate, with a birth weight of 2.34 kg (below the third percentile). The infant experienced aspiration pneumonia at the time of birth. The child was the firstborn of non-consanguineous parents of Han Chinese descent. The father was diagnosed with chronic hepatitis B, while the mother was identified as having hypothyroidism.

The parents denied a family history of other liver diseases or inherited metabolic disorders. A physical examination was conducted. The infant’s weight was 3.6 kg (<3rd percentile), height was 53 cm (<3rd percentile), head circumference was 37 cm ( 3rd to 10th percentile). The infant displayed a chubby face (Supplement Figure S1.B) and moderate jaundice of the skin. No enlargement of the liver or spleen was observed. The muscle strength and tone were found to be within the normal range for all limbs, as determined by clinical examination.

**Fig. 1:**
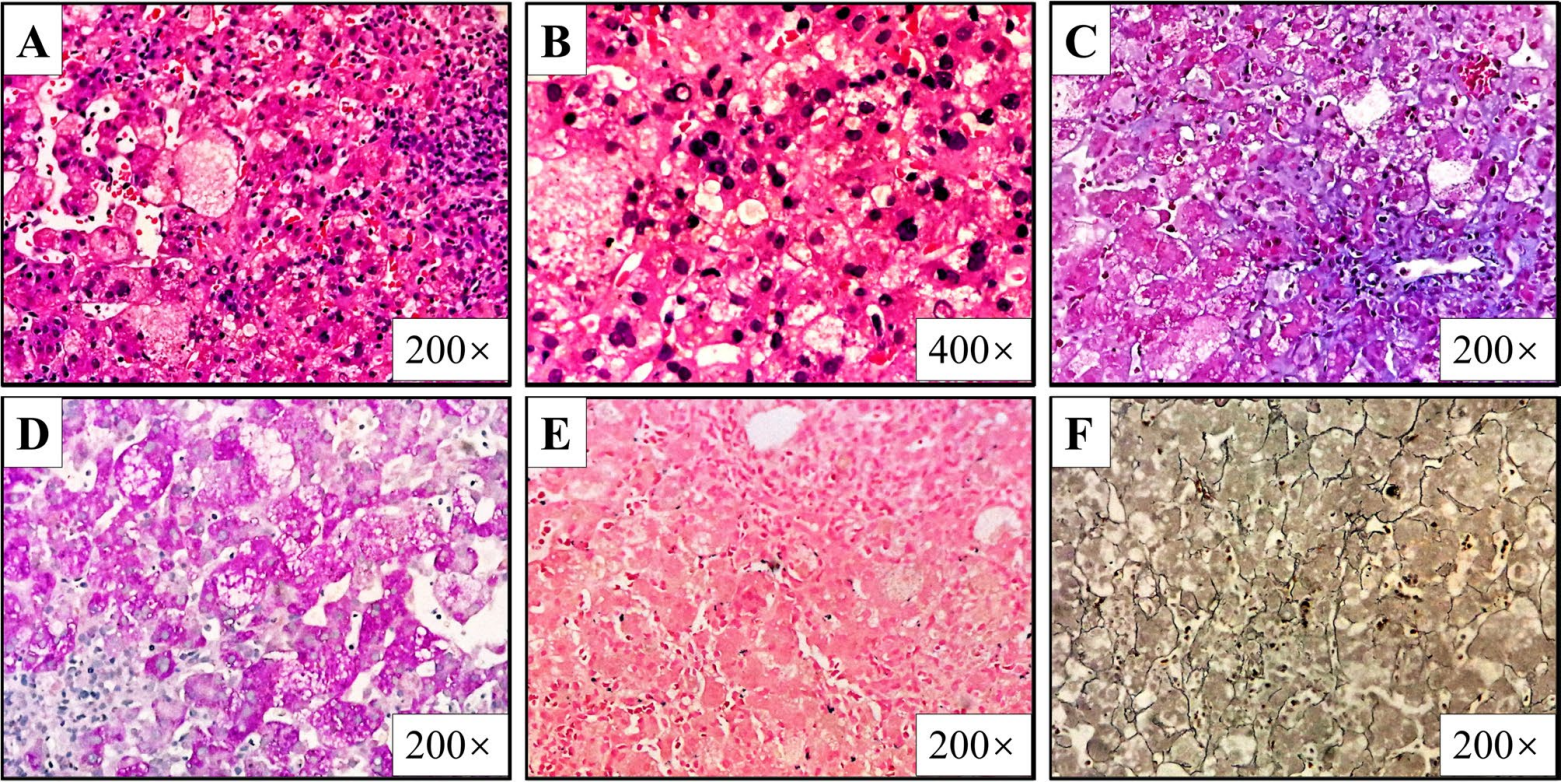
Histopathologic features, liver biopsy specimen, case 2. hepatocyte ballooning and the formation of multinucleated giant hepatocytes. Kupffer cells proliferated in the sinusoids of the liver. Fibrous tissue proliferated in the portal area and separated the hepatic lobules. Bridging fibrosis formation and bile duct hyperplasia were observed. A - F, respectively hematoxylin and eosin (**A**&**B**), periodic acid– Schiff technique (**C**), Masson’s trichrome stain (**D**), reticulin stain (**E**) and Perls’ stain for iron (**F**). For original magnifications, see individual images

The laboratory examination (Supplement Table S1) revealed the presence of anemia (hemoglobin: 98 g/L (NR, 99–196 g/L)) with elevated reticulocytes (4.3% (NR, 0.5–1.5%)). Biomarker values for the hepatobiliary system demonstrated elevated levels of TB (172.5umol/L (NR, 3.4-11.1umol/L)), DB (129.6umol/L (NR, 0-6umol/L)), ALT (310.8U/L (NR, 8-71U/L)), AST (630.0U/L (NR, 21-80U/L)), TBA (252.3U/L (NR, 0-10umol/L)), a progressive decline in GGT(173.4U/L (NR, 9-150U/L)) accompanied by hypoalbuminemia (27.5 g/L (NR, 35–50 g/L)), recurrent episodes of severe hypoglycemia (0.9mmol/L (NR, 3.9-6.1mmol/L)), mild abnormalities in coagulation (INR 1.4 (NR, 0.8–1.2), subsequent to vitamin K1 injection), elevated blood ammonia (99umol/L (NR, 18-72umol/L)), and elevated ferritin (703.1ng/mL (NR, 26-287ng/mL)). The post-corneal embryonic ring of the eye was found to be positive. The results of tests for Epstein-Barr virus by DNA-PCR, cytomegalovirus by DNA-PCR, and herpes simplex virus by DNA-PCR in blood were negative. Serum samples tested negative for HBsAg, anti-HBs, HBeAg, anti-HBe, anti-HBc, anti-HCV, and antibodies to syphilis and human immunodeficiency virus. Fecal enterovirus RNA, blood smears, blood tandem mass spectrometry, ANA, SMA, LKM-1, LC-1, AMA, morning cortisol, TSH, free T3, free T4, and direct and indirect Coombs tests were all within the normal range. Ultrasound examination revealed an abnormal texture of the liver parenchyma. Chest radiographs and full-length orthopantomograms of the spine found nothing outside the normal range. The results of hepatobiliary scintigraphy using Tc-99 m Mebrofenin indicate a moderate degree of uptake and impaired hepatobiliary excretion. Cardiac ultrasound demonstrated that the foramen ovale was not closed, with a diameter of 2.3 mm. Liver pathology (Fig. 1) demonstrated hepatocyte ballooning and the formation of multinucleated giant hepatocytes with a modified Scheuer score of G3S2. WES was conducted on the proband’s DNA sample at the Children’s Hospital of Fudan University, revealing the presence of biallelic variants in the *IARS1* gene, specifically [c.2354del (p. Lys785Arg fs*3)] was paternally inherited and [c.1556G > A (p. Arg519His)] was maternally inherited. The results were validated by Sanger sequencing.

The patient was diagnosed with IARS1 deficiency, cholestasis, anemia, hypoalbuminemia, coagulation abnormality and hypoglycemia. The patient was fed a lactose-free, high MCT formula, supplemented with albumin and fat-soluble vitamins, and subsequently discharged after demonstrating improvement. A second examination was conducted at the age of two years, revealing an Hb concentration of 111 g/L (NR, 112–149 g/L). Furthermore, the patient displayed elevated levels of ALT (91 U/L (NR, 7-30U/L)), AST (175 U/L (NR, 14-44U/L)), and GGT (110.2 U/L (NR, 5-19U/L)), concomitant with reduced albumin levels (38.2 g/L (NR, 40–54 g/L)). However, other indicators, including TB, DB, TBA, alpha-fetoprotein, ferritin, and coagulation function were within the normal range. The child was aged two and six months at the time of telephone follow-up. The patient’s weight was still<3rd percentile (10.8 kg), and height 3rd to 10th percentile (88 cm). Despite exhibiting the capacity to run and demonstrate typical cognitive abilities, he exhibited delayed linguistic development, with an expressive vocabulary of only two words.

#### Case 3

Case 3 is a 3.5-month-old male child who was admitted to the hospital with a two-month history of cough and a two-week history of shortness of breath. Since the age of 1.5 months, the subject has exhibited a recurrent cough. During this period, the patient also presented with vomiting, which led to a diagnosis of gastric torsion. A chest CT scan revealed evidence of pulmonary inflammation, while laboratory tests indicated the presence of cholestasis, anemia and hypoalbuminemia. Despite receiving treatment with a range of antibiotics at the local hospital, the patient’s condition deteriorated. This was evidenced by a worsening cough and the onset of shortness of breath, which occurred in the absence of any convulsions. This resulted in a transfer to our centre for further management. The infant had been delivered via caesarean section at 35 + 4 weeks’ gestation due to placental abruption, with a birth weight of 2.2 kg, which falls within the third to tenth percentile for gestational age. The infant was the first child of non-consanguineous parents of Han Chinese origin. The parents denied any family history of liver disease or inherited metabolic disorders. A physical examination was conducted. The infant’s weight was 4.3 kg (<3rd percentile), height was 58.5 cm (10th to 25th percentile), head circumference was 37.5 cm (<3rd percentile), and exhibited a breathing rate of 45 breaths per minute. Furthermore, the infant exhibited indications of malnutrition, a chubby face, pale lips and mouth, but no cyanosis around the lips. However, there was a slight shortness of breath, but no evidence of the triple concave sign. Moreover, the presence of sputum sounds in both lungs was evident. The abdomen was soft, with the lower edge of the liver situated at a distance of 3.0 cm from the right rib cage. The muscle strength and tone of the limbs were within the normal range.

The laboratory examination (Supplement Table S1) revealed the presence of anemia (hemoglobin: 62 g/L (NR, 99–196 g/L)), accompanied by elevated levels of leukocytes (32 × 10^9^/L (NR, 5–12 × 10^9^/L)), platelets (893 × 10^9^/L (NR, 100–300 × 10^9^/L)), and C-reactive protein (34 mg/L (NR, <8 mg/L)). Bone marrow cytological tests demonstrated markedly active myeloproliferation, with a predominantly granulomatous hyperplastic response. Biomarker values for the hepatobiliary system demonstrated a mild elevation in TB (55.9umol/L (NR, 3.4-11.1umol/L)), DB (43.8umol/L (NR, 0-6umol/L)), TBA (76.7umol/L (NR, 0-10umol/L)), ALT (48U/L (NR, 8-71U/L)) and AST (96U/L (NR, 21-80U/L)), while GGT exhibited a progressive decrease. Serum biochemistry revealed hypoalbuminemia (28.6 g/L (NR, 35–50 g/L)), hypoglycemia (2.3mmol/L (NR, 3.9-6.1mmol/L)), coagulation abnormalities (INR 2.1 (NR, 0.8–1.2) subsequent to vitamin K1 injection), a decrease in fibrinogen (0.4 g/L (NR, 2–4 g/L)), and an elevation in ferritin (719.2ng/mL (NR, 26-287ng/mL)). The blood ammonia level was within the normal range, while the level of IL-6 was 314.5pg/mL (NR,<16.6pg/mL), and β-1,3-D-glucan 101.7pg/mL(NR,< 60pg/mL). A sputum culture identified the presence of *Acinetobacter baumannii*. The alveolar lavage fluid was found to contain DNA of a *Mycoplasma* species. The galactomannan test, alveolar lavage fluid metagenomic next-generation sequencing, coagulation factor activity, ANA, SMA, LKM-1, LC-1, AMA, morning cortisol, TSH, free T3, free T4, blood tandem plasmapheresis, and urinary organic acids were all within the normal range. An enhanced CT of the chest (Supplement Figure S1. C-E) revealed extensive patchy shadows in both lungs, extensive lobular septal thickening in both lungs, and widening of the interlobar fissures. Periodic acid-Schiff staining of the alveolar lavage fluid yielded a positive result, with the unstructured material exhibiting a pink coloration. A computed tomography scan of the upper abdomen revealed an enlarged liver. Results of a portal vein enhancement CT and cardiac ultrasound were within the normal range. A liver biopsy showed vacuoles of varying sizes (macrovesicularity predominates) in most hepatocytes, and fibrotic tissue proliferation and partially separating the hepatic lobules in the ductal area. WES was conducted on the proband’s DNA sample at the Children’s Hospital of Fudan University, revealing biallelic variants in the *IARS1* gene, specifically [c.1330 C > T (p. Arg444X)], paternally inherited, and [c.443 A > G (p. Tyr148Cys)] that was maternally inherited. The results were corroborated by Sanger sequencing.

At the outset of the case, a diagnosis of diffuse parenchymal lung disease and liver disease was considered a possibility. Following the revelation of the genetic results, a diagnosis of IARS1 deficiency, pulmonary alveolar proteinosis (PAP), acute liver failure, respiratory failure, cholestasis, hypoalbuminemia, coagulation abnormality, hypoglycemia, growth retardation, gastroesophageal reflux disease, gastric torsion, anemia, and thrombocytosis was confirmed. The child was treated with a lactose-free, high MCT formula, a range of antibiotics including antifungal drugs, hormonal anti-inflammatory agents, bronchoscopic lavage therapy, ursodeoxycholic acid, erythrocyte suspension and a fresh frozen plasma infusion. The patient was discharged with an improved condition following this course of treatment. Subsequently, the patient was readmitted to the hospital on multiple occasions due to respiratory infections. Following the initial discharge, there was a gradual return to normal liver function. The patient’s hemoglobin levels were maintained at a range of 98–134 g/L (NR, 112–149 g/L), while their albumin levels remained within the 30–35.2 g/L range (NR, 40–54 g/L). Malnutrition and stunting were becoming increasingly evident. At the age of three years and one month, the child’s weight was<3rd percentile (9.5 kg), and height<3rd percentile (82 cm). The child was able to sit independently, vocalize the word “mummy”, and demonstrate recognition of individuals in his environment. However, he exhibited an inability to run and jump. Additionally, he exhibited indications of hypotonia. The child unfortunately succumbed to respiratory failure at about three years and two months old.

### Literature review

**Fig. 2:**
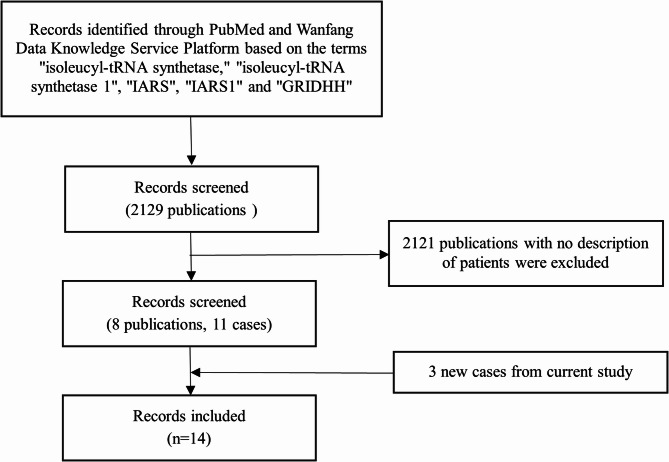
Diagram of screening and selection of included cases

A total of 2129 publications were subjected to analysis using the databases PubMed and Wanfang Data Knowledge Services Platform with search terms of “isoleucyl-tRNA synthetase”, “isoleucyl-tRNA synthetase 1”, “IARS”, “IARS1” and “GRIDHH”. The majority of these publications were not clinical studies. Studies lacking patient information were excluded, resulting in the inclusion of eight relevant publications (Fig. 2), including 11 previously reported cases and 3 new cases. A total of 14 cases were included in the research. The clinical history of each case was evaluated, including the patient’s country of origin, sex, age at the time of their last assessment, and vital status. Additionally, clinical features of the main organ systems involved were scrutinized according to Human Phenotype Ontology terminology. The data acquisition period concluded on 1 March 2024.

### Phenotype summary

**Fig. 3:**
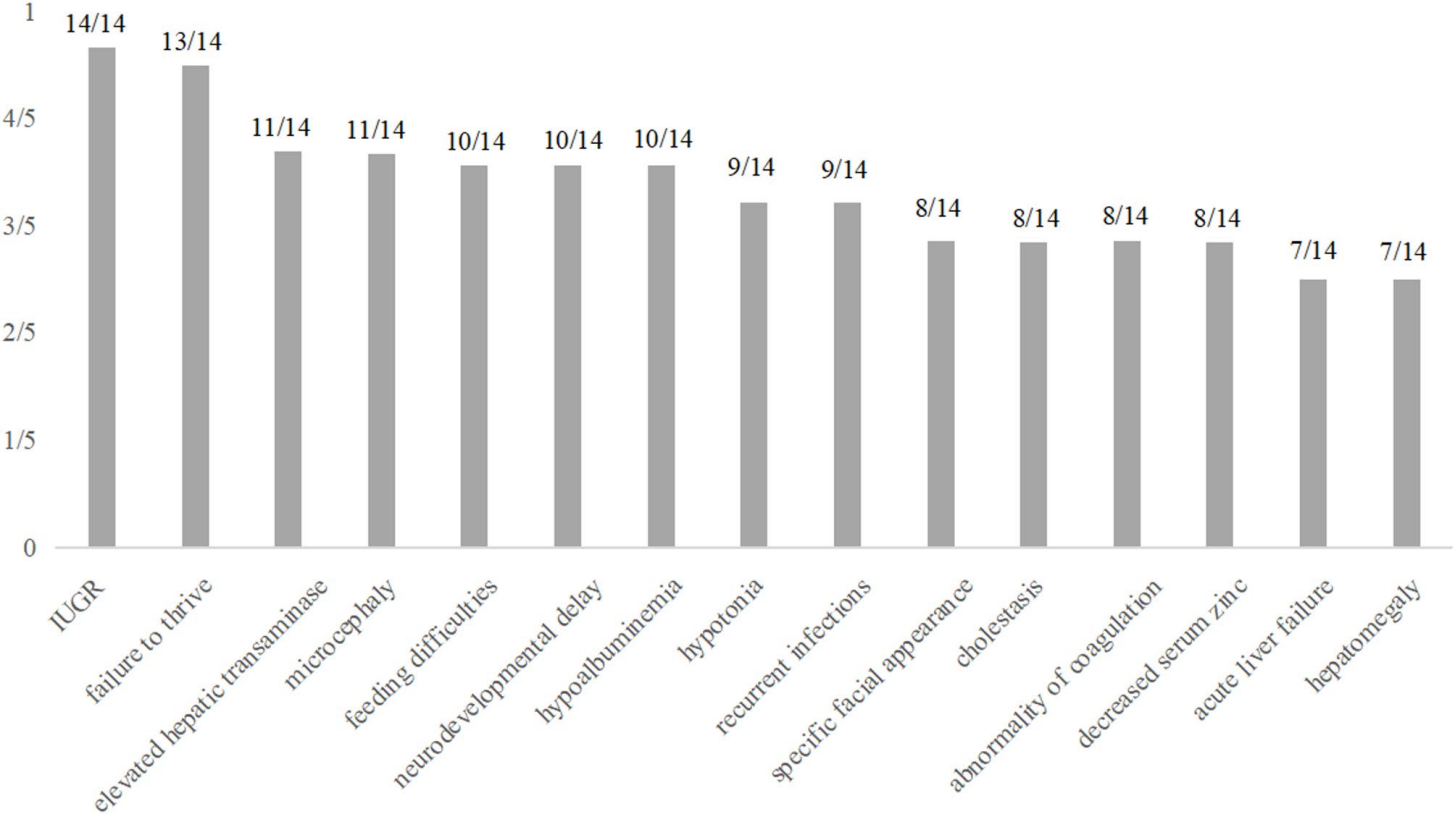
Most prevalent clinical findings in the study cohort. Most prevalent clinical signs within our cohort, present in at least ten patients and 50% of analysed individuals, including intrauterine growth retardation, failure to thrive, elevated hepatic aminotransferases, microcephaly, feeding difficulties, neurodevelopmental delay, hypoalbuminemia, hypotonia, recurrent infections and so on. IUGR, intrauterine growth retardation

A comprehensive description of the clinical features of all 14 cases, including the newly reported cases, was provided. The cases were categorized in accordance with the organ system(s) involved. Phenotypes with an incidence rate exceeding 50% were classified as common (Table 1; Fig. 3).

The cases comprised nine males and five females, with geographical origins in Asia (*n* = 9) and Europe (*n* = 4). Among the cases, one child was born prematurely, while the remaining 13 were born at full term (92.8%). Intrauterine growth retardation (IUGR) was present in all cases, and feeding difficulties were a prevalent postnatal phenotype observed in 10 out of 14 cases (71.4%). Despite the administration of nutritional supportive treatments, failure to thrive was observed in 13 of the 14 children by the time of last follow-up (92.8%).

Liver abnormalities were observed in 12 cases (85.7%), including elevated transaminase levels (11/14, 78.6%), cholestasis (8/14, 57.1%), liver failure (7/14, 50%), coagulation abnormalities (8/14, 57.1%), hyperammonemia (3/14, 21.4%), hypoglycemia (3/14, 21.4%), hepatomegaly (7/14, 50%) and splenomegaly (3/14, 21.4%). Neurological abnormalities were observed in all 14 cases (100%), including microcephaly (11/14, 78.6%), neurodevelopmental delay (10/14, 71.4%), hypotonia (9/14, 64.3%), delayed speech development (6/14, 42.8%), epilepsy (2/14, 18.2%), hearing abnormalities (2/14, 18.2%) and joint laxity (2/14, 18.2%). Individuals older than five years of age were also evaluated, and it was found that six of the seven cases exhibited impaired intellectual development, representing a prevalence of 85.7%.

In addition, hypoalbuminemia (10/14, 71.4%), recurrent infections (9/14, 64.3%), special facial features (8/14, 57.1%), zinc deficiency (4/7, 57.1%), anemia (5/14, 35.7%) and PAP (3/11, 21.4%) were observed. In the third case, which has only recently been reported, gastric torsion was also observed in infancy, a phenomenon that has not previously been documented.

At the time of the last follow-up, the age of the 14 children ranged from 3 months to 19 years. Of the 14 children, nine survived, while five died, resulting in a mortality rate of 35.7%. Two children died of acute liver failure and respiratory failure at 3 and 4 months of age, respectively. Two children succumbed to respiratory failure at 3 years and 1 month of age and 6 years of age, respectively, while one child died of acute liver failure at 19 months of age.

### Pathological findings

Two of the three new cases underwent liver biopsy, with specimens obtained by needle biopsy from cases 2 and 3. Of the 14 reported cases, pathological findings were available for eight patients aged between one month and nine years old. The most frequently observed features were fibrosis (6/8, 75%), hepatocellular steatosis (5/8, 62.5%), cholestasis (5/8, 62.5%), biliary hyperplasia (2/8, 25%), hepatocellular oedema (1/8, 12.5%), fusion of hepatocytes into multinucleated hepatic giant cells (1/8, 12.5%), and unremarkable inflammation.

### Genetic characterization

Six novel variants in *IARS1* were identified in three new patients, including c.2017–2 A > G (splicing), c.241 A > G (p. Arg81Gly), c.2354del (p. Lys78Arg fs*3), c.1556G > A (p. Arg519His), c.1330 C > T (p. Arg444Ter) and c.443 A > G (p. Tyr148Cys). No other plausible candidate variants consistent with the patient’s clinical phenotype and recessive (autosomal or X-linked) inheritance were identified. The pathogenicity of the newly discovered missense *IARS1* variants was evaluated using in silico tools, as detailed in Table 2. Of these, c.241 A > G, c.443 A > G, c.1330 C > T, and c.1556G > A are situated within the class I functional domain of the synthetase, whereas c.2354del is located within the anticodon binding domain of the tRNA ligase. The results of the conservation analyses demonstrated that p. Arg81, p. Tyr148, p. Arg519, and p. Lys785 are highly evolutionarily conserved in mammals. A total of 24 variants were identified in the 14 cases, comprising frameshift variant (*n* = 3), nonsense variant (*n* = 3), splice variant (*n* = 2), and missense variant (*n* = 16). The locations of all these variants are illustrated in Supplemental Figure S2. No special findings were found in the distribution. With limited case numbers, the relationship between genotype and phenotype was not evaluated.

**Table 2:** Allele frequencies and in Silico prediction of 6 novel I*ARS1* variants

cDNA change (NM_020117.11)	Protein change (NP_000383.2)	gnomAD	gnomAD_EAS	MuT	SIFT	M-CAP	REVEL	Polyphen2	GERP	SpliceAI	ClinPred	AlphaMissense	LRT
c.[2017–2 A > G];	p.[splicing]	-	-	D	-	-	-	-	D	T	-	/	-
c.[241 A > G];	p.[Arg81Gly]	-	-	D	D	D	LD	LD	D	/	D	D	D
c.[2354del]	p.[Lys785Argfs*3]	0.00003185	0.00064185	-	-	-	-	-	-	/	-	-	-
c.[1556G > A]	p.[Arg519His]	-	-	D	D	D	LD	LD	D	/	D	D	D
c.[1330 C>T]	p.[Arg444X]	-	-	D	-	-	-	-	D	/	-	-	D
c.[443 A > G]	p.[Tyr148Cys]	0.00003185	0	D	D	D	D	LD	D	/	D	D	D

Abbreviations:

gnomAD and gnomAD_EAS, allele frequencies of corresponding variants in all populations and in East Asian populations in gnomAD (http://gnomad-old.broadinstitute.org/) respectively;

-: not reported; D: disease-causing; LD: likely disease-causing; T:Affects splicing; /:not applicable; T: Tolerated;

MuT, MutationTaster (http://www.mutationtaster.org);

SIFT, Sorting Intolerant From Tolerant (http://provean.jcvi.org/index.php);

M- CAP, Mendelian Clinically Applicable Pathogenicity (http://bejerano.stanford.edu/MCAP/);

REVEL, Rare Exome Variant Ensemble Learner (https://sites.google.com/site/revelgenomics/);

PolyPhen2 (Polymorphism Phenotyping v2), http://genetics.bwh.harvard.edu/pph2/index.shtml;

GERP (Genomic Evolutionary Rate Profiling), http://mendel.stanford.edu/SidowLab/downloads/gerp/;

SpliceAI, (https://spliceailookup.broadinstitute.org/);

ClinPred, (https://sites.google.com/site/clinpred/);

AlphaMissense, (https://github.com/deepmind/alphamissense);

LRT (Likelihood Ratio Test), (http://www.genetics.wustl.edu/jflab/lrt_query);

### Statistical analysis

**Fig. 4:**
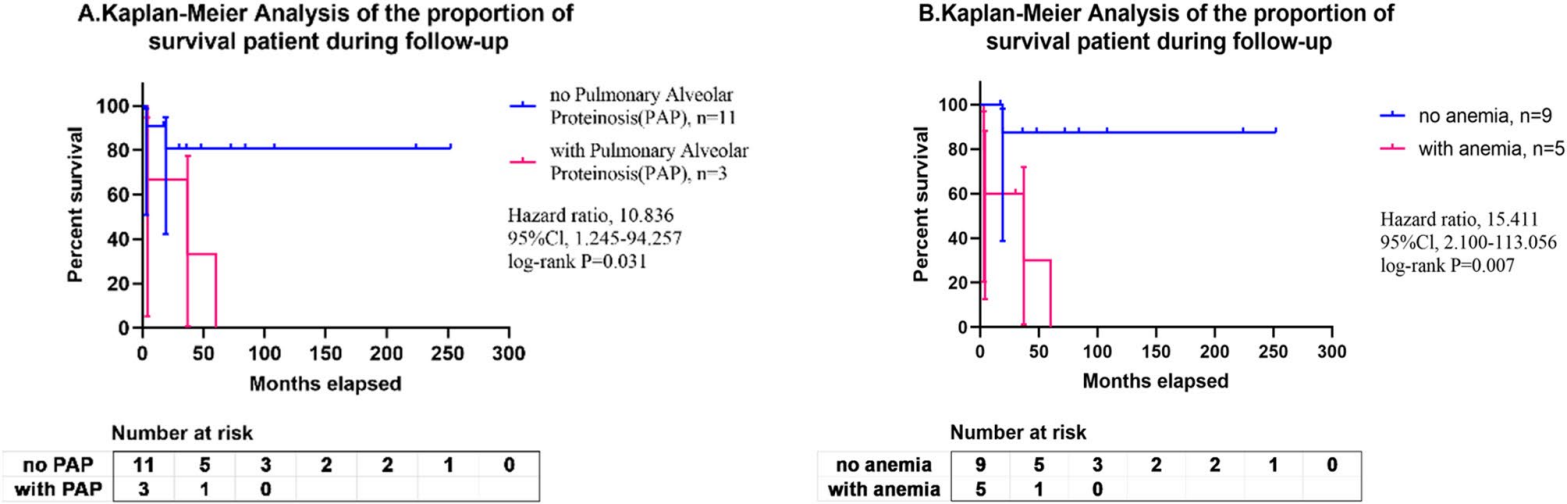
Kaplan-Meier plotter analysis: (**A**) PAP and (**B**) anemia were associated with a poor prognosis (death)

Fisher’s exact test and Kaplan-Meier plots were employed to conduct survival analyses and evaluate the correlation between patients’ demographic characteristics, including the child’s gender, and the primary clinical manifestations. The principal clinical manifestations under consideration were liver failure, PAP and anemia. The follow-up data for the patients was examined, including their age at death, the duration of the follow-up period, and the date of their last clinical contact. Fisher’s exact test showed that the occurrence of PAP (*P* = 0.027) and anemia (*P* = 0.022) were significantly correlated with a prognosis of death (Supplemental Table S2). The results of Kaplan-Meier plots demonstrated no significant correlation between patient prognosis and clinical course, except for the occurrence of PAP (HR = 10.837, 95% CI = 1.246–94.257, *P* = 0.031) and anemia (HR = 15.411, 95%CI = 2.101-113.057, *P* = 0.007) (Supplemental Table S3, Fig. 4).

## Discussion

Three new cases of IARS1 deficiency have been documented, representing an expansion of the pool of medical records by approximately 30%. In total, 14 cases were summarized, comprising the 11 previously reported cases and the 3 additional cases presented herein. The disease involves multiple systems, exhibiting diverse clinical phenotypes. The severity of the disease is quite variable, with five of 14 patients succumbing to it. The primary causes of mortality were respiratory failure and liver failure.

The liver, as a highly metabolically active organ, is significantly affected by IARS1 deficiency, exhibiting a wide range of phenotypes, including recurrent liver failure. Half of the cases had experienced at least one episode of liver failure, with half of these cases resulting in mortality. This suggests that IARS1 deficiency may be a potential cause of acute liver failure in infants and children, with likely fatal consequences. Most liver failure occurred within the first year of life, however, and none occurred after two years of age, indicating that younger age may be associated with an elevated risk of liver failure. A review of follow-up data from previous cases revealed that certain phenotypes, such as elevated transaminases and hypoalbuminemia, demonstrated improvement with age, returning to normal levels in school age children. This suggests that the prognosis for liver function is favorable following a period of peak growth and infection in early life. Liver biopsies were conducted in 8 cases, with an age range of one month to nine years. The principal pathological features are fibrosis in the confluent area, steatosis and cholestasis, with no evidence of inflammation.

Much like other deficiencies associated with ARSs, IARS1 deficiency has been linked to a range of neurological disorders, with notable phenotypic variation observed. The principal characteristics encompass a spectrum of neurodevelopmental delays, frequently accompanied by microcephaly. A recent case report documents the occurrence of developmental regression in a previously unreported instance [10]. Nevertheless, despite the implementation of an intensive rehabilitation program, children may still experience a mild to severe intellectual disability over time. The extent of neurological involvement may be a significant determinant of long-term prognosis and quality of life.

Of the 14 children, five succumbed to the disease, indicating a poor prognosis. In the present study, Fisher’s exact test and Kaplan-Meier plots were employed to evaluate the primary factors that may be associated with prognosis. We found that the occurrence of PAP and anemia are the primary factors associated with a poor prognosis. It is advised that children who present with both PAP and anemia receive priority attention. Furthermore, it was observed that half of the children who developed liver failure died, which led to the hypothesis that liver failure may be a risk factor. Nevertheless, no statistically significant difference was identified in the analysis. This may be attributed to the limited number of cases. It is anticipated that with an expansion of the sample size, further risk factors of the disease may be identified, which could inform improvements in the therapeutic regimen and prognosis.

At present, there is no specific treatment available for the disease. The primary objective of treatment is to provide symptomatic relief. Sabin et al.. proposed the administration of the appropriate amino acid for each specific ARS deficiency as a potential treatment [6]. This approach has been supported by several studies [3, 12, 13], which have demonstrated promising therapeutic results. Treatment with isoleucine for IARS1 deficiency may prove to be an effective and specific intervention. Nevertheless, further prospective studies with larger samples are required to confirm this conclusion. Furthermore, a high-protein diet may prove beneficial in alleviating symptoms, a strategy analogous to that proposed by Casey et al. for leucyl-tRNA synthase 1 deficiency [14]. It is recommended that an intake of at least 2.5 g/kg of protein be administered either enterally or parenterally. It is nevertheless recommended to exercise caution when contemplating protein intake in patients with IARS1 deficiency and liver failure.

*The main limitation of this study is the small sample size of the retrospective analysis*,* which included just 14 patients. Further prospective studies involving more cases are needed to improve our understanding of the risk factors that affect the prognosis of IARS defects.*

## Conclusions

This study presents three new cases of IARS1 deficiency and offers a comprehensive summary of the clinical features, pathological findings, and molecular genetic characteristics observed in these cases. Furthermore, our findings suggest that the presence of PAP and anemia may be associated with a poor prognosis.

## Electronic supplementary material

Below is the link to the electronic supplementary material.

Supplementary Material 1

Supplementary Material 2

Supplementary Material 3

Supplementary Material 4

Supplementary Material 5

## Data Availability

All data generated or analysed during this study are included in this published article [and its supplementary information files].

## Abbreviations

ALT: Alanine aminotransferase
ARSs: Aminoacyl-tRNA synthetases
AST: Aspartate aminotransferase
DB: Direct bilirubin
GGT: Gamma-glutamyl transferase
GRIDHH: Growth retardation, impaired intellectual development, hypotonia, and hepatopathy
IARS1: Isoleucyl-tRNA Synthetase 1
INR: International normalized ratio
IQR: Interquartile range
IUGR: Intrauterine growth retardation
MCT: Medium-chain triglyceride
NR: Normal range
PAP: Pulmonary alveolar proteinosis
TB: Total bilirubin
TBA: Total bile acid
WES: Whole exome sequencing
