# Supplementary material for: Pulmonary alveolar proteinosis and anemia may be associated with poor prognosis in patients with IARS1 variants
Source: Orphanet J Rare Dis. 2025 Jul 9;20:350. doi: 10.1186/s13023-025-03885-z (PMC12243253; doi:10.1186/s13023-025-03885-z)
Supplement: Supplementary file 1 — Supplementary Material 1 [file 13023_2025_3885_MOESM1_ESM.docx]

**
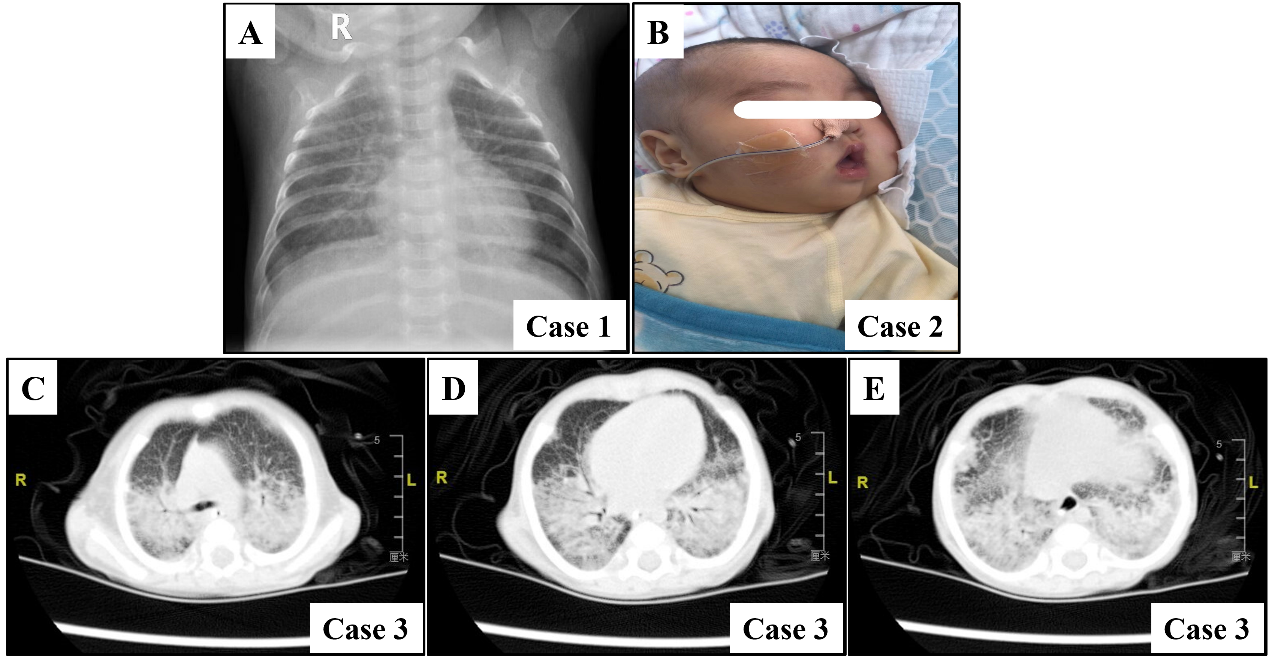
**

**Supplemental Figure 1**. The chest X-ray of case 1 showed increased and blurred texture in both lungs, interstitial changes were seen, and more flocculent and blurred shadows were seen in the middle and inner bands (A). Physical examination of case 2 indicated a chubby face (B). The CT scan of the lungs of case 3 showed extensive patchy shadows in the two lungs and extensive interlobular thickening in both lungs (C-E).
