# Supplementary figures and images for: Pulmonary alveolar proteinosis and anemia may be associated with poor prognosis in patients with IARS1 variants
Source: Orphanet J Rare Dis. 2025 Jul 9;20:350. doi: 10.1186/s13023-025-03885-z (PMC12243253; doi:10.1186/s13023-025-03885-z)

**
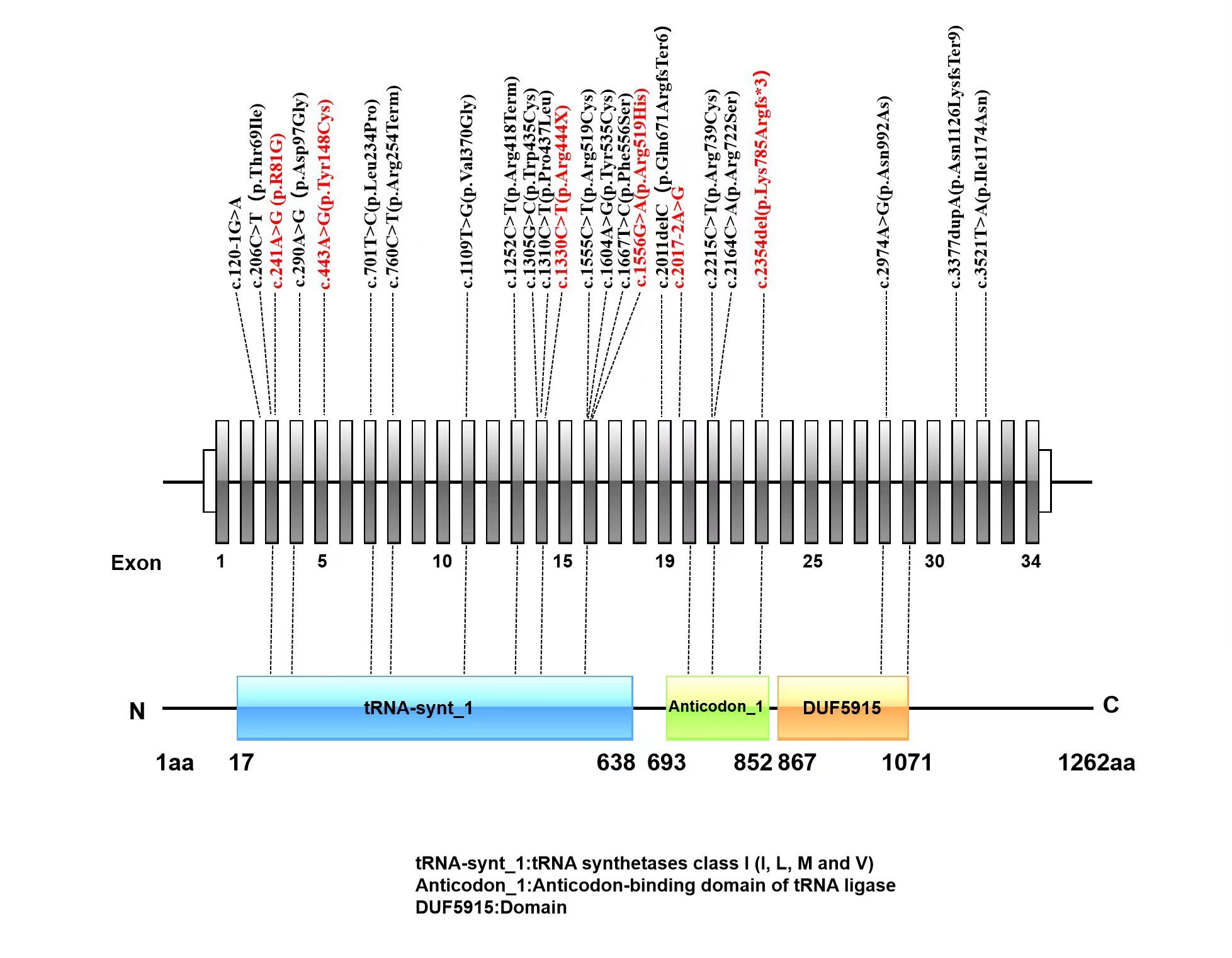
**

**Supplemental Figure 2**.Localization of *IARS1* variants in the study cohort.

Supplement: Supplementary file 2 — Supplementary Material 2 [file 13023_2025_3885_MOESM2_ESM.docx]
