# Supplementary material for: Pulmonary alveolar proteinosis and anemia may be associated with poor prognosis in patients with IARS1 variants
Source: Orphanet J Rare Dis. 2025 Jul 9;20:350. doi: 10.1186/s13023-025-03885-z (PMC12243253; doi:10.1186/s13023-025-03885-z)
Supplement: Supplementary file 3 — Supplementary Material 3 [file 13023_2025_3885_MOESM3_ESM.docx]

Supplement table S1. Results, laboratory testing of the 3 new cases

|  | P1 | P2 | P3 | NR |
| --- | --- | --- | --- | --- |
| Age | 2.5 m | 2.0 m | 3.5 m |  |
| WBC (×10^9^/L) | 8.1-17.4 | 7.1-11.0 | 13.4-32.0 | 5.0-12.0 |
| Hb (g/L) | 72.0-100.0 | 98.0-110.0 | 62.0-119.0 | 99.0-196.0 |
| MCV (fL) | 95.1-98.8 | - | - | 73.0-105.0 |
| MCH (pg) | 32.0-32.0 | - | - | 24.0-37.0 |
| MCHC (g/L) | 324.0-346.0 | - | - | 305.0-361.0 |
| PLT (×10^12^/L) | 107.0-159.0 | 298.0-323.0 | 269.0-893.0 | 100.0-300.0 |
| RET (%) | 3.8 | 4.3 | 3.8 | 0.5-1.5 |
| Alb (g/L) | 25.4-38.9 | 27.5-34.7 | 28.6-36.5 | 35.0-50.0 |
| ALT (U/L) | 43.0-66.0 | 206.3-310.8 | 21.0-48.0 | 8.0-71.0 |
| AST (U/L) | 76.0-109.0 | 370.0-630.0 | 51.0-96.0 | 21.0-80.0 |
| GGT (U/L) | 138.0-79.0 | 173.4 | 230.0-114.0 | 9.0-150.0 |
| TB (μmol/L) | 72.4-116.0 | 109.0-172.5 | 22.4-55.9 | 3.4-17.1 |
| DB (μmol/L) | 59.2-103.1 | 98.3-129.6 | 17.8-43.8 | 0.0-6.0 |
| TBA (μmol/L) | 144.4-380.4 | 143.4-252.3 | 18.7-76.7 | 0.0-10.0 |
| CK (U/L) | - | - | 32.0 | 24.0-170.0 |
| INR | 1.6-2.4 | 1.1-1.4 | 1.1-2.1 | 0.8-1.2 |
| Fibrinogen(g/L) | 0.5-1.0 | 1.6-1.8 | 0.4-5.1 | 2.0-4.0 |
| Ammonia (μmol/L) | 24.4-58.2 | 99.0 | 76.0 | 18.0-72.0 |
| Glycemia (mmol/L) | 1.6-2.4 | 0.9-7.0 | 2.3-3.7 | 3.9-6.1 |
| Ferritin (ng/mL) | - | 703.1 | 719.2 | 26.0-287.0 |

Abbreviations: Alb, albumin; ALT, alanine aminotransferase; AST, aspartate aminotransferase; CK, creatine kinase; DB, direct bilirubin; GGT, gamma-glutamyltransferase; Hb, hemoglobin; INR, international normalized ratio; MCH, mean corpuscular hemoglobin; MCHC, mean corpuscular hemoglobin concentration; MCV, mean corpuscular volume; NR, normal range; P, patient; PLT, platelets; RET, percentage of reticulocyte; TB, total bilirubin; TBA, total bile acid; WBC, white blood cells; -, not available.
