# Supplementary material for: Pulmonary alveolar proteinosis and anemia may be associated with poor prognosis in patients with IARS1 variants
Source: Orphanet J Rare Dis. 2025 Jul 9;20:350. doi: 10.1186/s13023-025-03885-z (PMC12243253; doi:10.1186/s13023-025-03885-z)
Supplement: Supplementary file 4 — Supplementary Material 4 [file 13023_2025_3885_MOESM4_ESM.docx]

Supplemental table S2. Fisher's exact test for the prognosis of IARS1 patients with or without PAP, anemia and ALF.

| Variable | Survive (n=9) | Dead (n=5) | P-value |
| --- | --- | --- | --- |
| with PAP group | 0 | 3 | 0.027 |
| no PAP group | 9 | 2 |  |
| with anemia | 1 | 4 | 0.022 |
| no anemia | 8 | 1 |  |
| with ALF group | 3 | 4 | 0.265 |
| no ALF group | 6 | 1 |  |

ALF: acute liver failure; PAP: pulmonary alveolar proteinosis.
