# Supplementary material for: Pulmonary alveolar proteinosis and anemia may be associated with poor prognosis in patients with IARS1 variants
Source: Orphanet J Rare Dis. 2025 Jul 9;20:350. doi: 10.1186/s13023-025-03885-z (PMC12243253; doi:10.1186/s13023-025-03885-z)
Supplement: Supplementary file 5 — Supplementary Material 5 [file 13023_2025_3885_MOESM5_ESM.docx]

Supplemental table S3. Statistical results of Kaplan-Meier survival analyses

| Item | HR (95%CI) | P（Log-rank test） |
| --- | --- | --- |
| Female vs Male | 1.454（0.219-9.634） | 0.698 |
| PAP vs without PAP | 10.837（1.246-94.257） | 0.031 |
| anemia vs without anemia | 15.411（2.101-113.057） | 0.007 |
| ALF vs without ALF | 6.196（0.972-39.494） | 0.053 |

ALF: acute liver failure; PAP: pulmonary alveolar proteinosis.
